# COVID-19 containment measures impact utilization and provision of healthcare in Europe: Julia Dratva

**DOI:** 10.1093/eurpub/ckac129.472

**Published:** 2022-10-25

**Authors:** J Dratva, A Klein, S Marti, F Wieber

**Affiliations:** 1 Institute of Public Health, Zürich University of Applied Sciences, Winterthur, Switzerland; 2 Medical Faculty, University of Basel, Basel, Switzerland

## Abstract

**Background:**

The COVID-19 containment measures, implemented to curb the pandemic, impacted health of children and adolescents by numerous pathways. We present the impact on health care utilization and provision.

**Methods:**

A systematic review on secondary health impact is ongoing (PubMed, PsychInfo, Embase). Literature is screened (title, abstract, full-text) by 2 researchers, and data of interest extracted systematically.. Inclusion criteria are age (0 - 25 yrs.), exposure: containment measures, outcome: secondary health outcome, and European data origin.

**Results:**

Jan. 2020 - Aug. 2021 10112 studies were identified, 337 were included. n = 60 were on health care utilization and provision. Utilization studies relied on objective hospital or registry data, care provision studies more often on survey data (professionals, parents). Data yields a large but varying decrease in emergency department visits during the lockdown: Italy ∼75%, Spain ∼65%, France ∼60%, and Germany ∼64%, and a substantial change in case mix and severity compared to comparable pre-COVID. Specialized and primary pediatric practices report that elective interventions were postponed, state of the art diagnostics withheld, and rehabilitation services disrupted. Vaccinations in infants, children, and adolescents dropped during the lockdown inversely proportional to children's age. Studies repeatedly suggest patients’ health services avoidance out of fear of infection and stay-at-home rules.Results on catch-up utilization and provision to follow (ongoing study).

**Conclusions:**

COVID-19 measures exerted a measurable impact on health utilization and provision in children and adolescents. The utilization was comparatively lower and service provision disrupted across Europe. So far little can be said about a potential recovery in terms of catch-up of visits, diagnostics, or treatments. Analyses of the long-term health impact of the observed effects is recommended and can serve to improve future pandemic preparedness.

**Key messages:**

• COVID-19 confinement measures had measurable secondary health impact on children and adolescents.

• Data on catch-up healthcare is important to establish long term impact and learnings.

